# Acute levodopa dosing around-the-clock ameliorates REM sleep without atonia in hemiparkinsonian rats

**DOI:** 10.1038/s41531-019-0096-2

**Published:** 2019-11-29

**Authors:** Vishakh Iyer, Quynh Vo, Anthony Mell, Siven Chinniah, Ashley Zenerovitz, Kala Venkiteswaran, Allen R. Kunselman, Jidong Fang, Thyagarajan Subramanian

**Affiliations:** 1 Program in Neuroscience, Department of Psychological and Brain Sciences, Indiana University, Bloomington, IN USA; 2 Department of Neurology, West Virginia University School of Medicine, Morgantown, WV USA; 3 Department of Neurology and Neural and Behavioral Sciences, The Pennsylvania State University College of Medicine, Hershey, PA USA; 4 Department of Public Health Sciences, The Pennsylvania State University College of Medicine, Hershey, PA USA; 5 Department of Psychiatry, The Pennsylvania State University College of Medicine, Hershey, PA USA

**Keywords:** Parkinson's disease, Circadian rhythms and sleep

## Abstract

Rapid-eye-movement (REM) sleep without atonia (RSWA), a marker of REM sleep behavior disorder (RBD), is frequently comorbid with Parkinson’s disease (PD). Although rodent models are commonly used for studying PD, the neurobiological and behavioral correlates of RBD remain poorly understood. Therefore, we developed a behavior-based criteria to identify RSWA in the hemiparkinsonian rat model of PD. Video recordings of rats were analyzed, to develop a criteria consisting of behavioral signs that occurred during polysomnographically confirmed epochs of sleep-wake stages. The *sleep-slouch*, a postural shift of the body or head caused only by gravity, was identified as a unique behavioral sign of REM sleep onset and was altered in hemiparkinsonian rats during RSWA. There was a significant correlation between the behavior-based criteria and polysomnograms for all sleep-wake stages in control but not hemiparkinsonian rats indicating a deterioration of sleep-wake architecture in parkinsonism. We then tested the efficacy of levodopa in ameliorating RSWA using intermittent and around-the-clock (ATC) dosing regimens. ATC levodopa dosing at 4 mg/kg for 48 h caused a significant reduction of RSWA as measured by polysomnography and the behavioral-based criteria along with an amelioration of forelimb motor deficits. Our findings show that the phenomenological correlates of RSWA can be reliably characterized in the hemiparkinsonian rat model. ATC levodopa administration ameliorates RSWA in this model without deleterious consequences to the overall sleep-wake architecture and therapeutic benefits for parkinsonian motor deficits. These findings suggest that further study may allow for the application of a similar approach to treat RBD in PD patients.

## Introduction

Disturbances of sleep are well documented in Parkinson’s disease (PD) and are estimated to occur in 60–90% of all PD patients.^1–4^ Rapid-eye-movement sleep behavior disorder (RBD) is one of the most common and disabling sleep disturbances associated with PD and is characterized by a loss of muscle atonia during rapid-eye-movement (REM) sleep and dream enactments.^5–8^ Therefore, the occurrence of REM sleep without atonia (RSWA) is considered the prototypical marker of RBD.^9^ PD patients with RBD report significant morbidity due to fatigue, excessive daytime sleepiness, injuries related to dream enactments, and fluctuating efficacy of PD therapeutics.^10–13^ Studies have also shown that the morbidity of sleep dysfunction significantly affects the quality of life of PD patients and may exceed the impact of the motor impairment and other complications.^10,14^ Finally, RBD symptoms frequently predate the diagnosis of PD motor symptoms and has been investigated as a putative biomarker for pre-clinical PD.^4,5,15–18^ Taken together, these factors underscore the need for a pre-clinical model that recapitulates the phenomenology of RSWA in PD and help close the major gap in this area of unmet therapeutic need.^14,19^

Although rodents are commonly used to model PD, few studies have investigated sleep dysfunctions in these pre-clinical models. Current pre-clinical models of sleep disorders in rodents utilize a combination of electroencephalography (EEG) and electromyography (EMG) to classify sleep-wake stages. Rodent sleep is typically divided into three principal categories: awake, REM sleep, and non-REM sleep (NREM).^20,21^ Unlike human polysomnography, most rodent sleep studies do not make use of time-locked video recordings or well-delineated behavior-based criteria for identifying sleep stages or its dysfunctions. Rodent sleep architecture also lacks many of the characteristic hallmarks commonly found in human sleep recordings. Therefore, rodent sleep recordings present a unique challenge in identifying sleep disorders that are associated with movement such as RBD, as EMG becomes unreliable as a marker for RBD-associated movement. Previously, we have shown that the sleep architecture in the unilateral 6-hydroxydopamine (6-OHDA) lesioned hemiparkinsonian rat model of PD, an animal model that has contributed to the development of most of the medications in current use for PD, resembles that of human PD patients with RBD.^22^ Here we demonstrate that this animal model exhibits RBD like behavioral features and validated a set of behavior-based criteria for sleep-wake stages that is correlated with rat sleep architecture to reliably assess RSWA.

Most treatments currently available for RBD in PD patients like clonazepam and melatonin are considered suboptimal due to their variable efficacy and side effects such as sedation, motor incoordination, confusion, memory dysfunction etc.^23^ In contrast, levodopa one of the most commonly prescribed anti-PD medication has been shown to be effective in ameliorating RBD in several studies especially when RBD precedes the onset of the motor symptoms of PD.^14,24,25^ The effects of levodopa in patients who only present with RBD and do not develop PD in their lifetime is not discussed here. In such patients, levodopa did not meet evidence-based data to be recommended as a treatment.^23^ All current PD patients, however, will receive levodopa in their lifetime and exposure to this medication is unavoidable in contemporary medicine as it is by far the most effective pharmacotherapy for PD. Unfortunately, long-term use of intermittent oral levodopa causes disabling motor complications in most PD patients. A recent review of clinical trials, however, has shown that levodopa dosing, administration route, and regimen may be critical in affecting the onset of these medication-induced complications and novel dosing regimens with strict compliance may be able to overcome their side effects.^26^

The advent of extended-release oral levodopa formulations and intrajejunal pump-based levodopa administration methods have permitted longer and more continuous dosing regimens. Such treatments with continuous or around-the-clock (ATC) dosing may help avoid the medication-induced complications of levodopa. However, the efficacy of such dosing regimens remains largely unexplored in pre-clinical models of PD. Therefore, we investigated whether RSWA in the 6-OHDA hemiparkinsonian rodent model of PD is responsive to levodopa therapy when administered ATC. Our results indicate that ATC dosing of levodopa mitigates both motor symptoms like forelimb bradykinesia and non-motor symptoms such as RSWA without affecting the overall architecture of sleep-wake stages in this rat model. Our findings suggest that such an ATC dosing regimen with continuous delivery of levodopa could be a potential experimental therapeutic strategy for RBD in PD patients.

## Results

### ATC levodopa dosing leads to accurate classification of hemiparkinsonian rats comparable to unlesioned controls

**Fig. 1:**
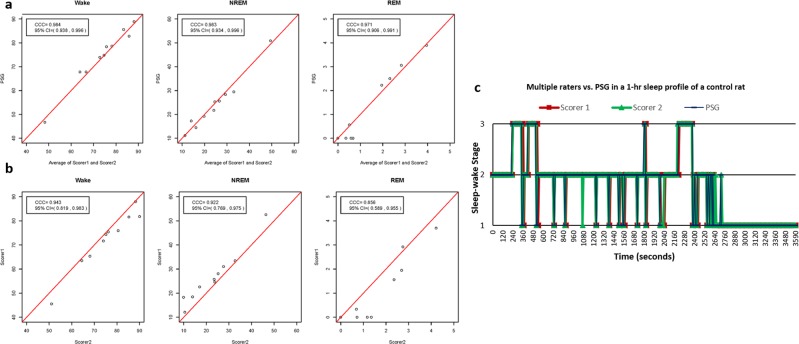
Comparison of sleep-wake stage classification between polysomnography and the behavior-based criteria and between two separate blinded raters in control rats. **a** In control rats (*n* = 10), there was good agreement between the average of the raters (Average of Scorer 1 and Scorer 2) and polysomnography (PSG) indicating the high face validity of the behavior-based criteria for classification of all sleep-wake stages (Wake, NREM, and REM). **b** Comparisons between raters (Scorer 1 vs. Scorer 2) also showed a good level of agreement indicating high inter-rater reliability of the behavior-based criteria. **c** A 1-h graphed sleep profile comparing classification between the raters and PSG also showed close matches in all sleep-wake stages, with all REM periods correctly identified by the raters.

**Fig. 2:**
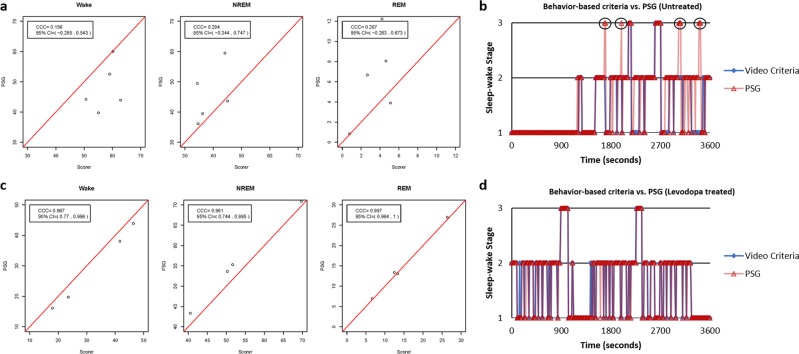
Comparison of sleep-wake stage classification between untreated and levodopa-treated groups using the behavior-based criteria and polysomnography in hemiparkinsonian rats. **a** In untreated hemiparkinsonian rats (*n* = 5), there was poor agreement between the behavior-based criteria compared with polysomnography (PSG) for all sleep-wake stages, which was indicative of the decline in quality of the behaviors used to apply the criteria. **b** A 1-h graphed sleep profile comparing classification between the raters and the polysomnograms showed several false-positive and false-negative identifications of REM periods by either or both raters (circled). **c** ATC levodopa at 4 mg/kg in treated hemiparkinsonian rats (*n* = 4) helped alleviate the adverse behavioral changes caused by lesioning as indicated by the good levels of agreement between the behavior-based criteria compared with PSG for all sleep-wake stages post levodopa treatment. **d** A 1-h graphed sleep profile in a levodopa-treated hemiparkisonian rat also showed close matches in all sleep-wake stages similar to that of a control rat.

The accuracy of the behavior-based criteria proposed here was tested by comparing the average scores of the two independent blinded raters with polysomnography (PSG) recordings for all sleep-wake stage (Wake, NREM, and REM) classification. To compute the inter-rater reliability of the criteria, classification was also compared between the two raters for all sleep-wake stages. In unlesioned control rats, the sleep-wake stage classification using the behavior-based criteria (Average of Scorer 1 and Scorer 2) showed a good agreement (concordance correlation coefficient (CCC) > 0.8) compared with PSG for all sleep-wake stages (Fig. 1a, Table 1). The inter-rater reliability of the criteria also showed good agreement between the two raters (CCC >0.8) for all sleep-wake stages (Fig. 1b, Table 1). Graphed sleep profiles between the raters and PSG also showed close matches in the sleep-wake stage classification (Fig. 1c). In untreated hemiparkinsonian rats, there was poor agreement (CCC <0.8) between the behavior-based criteria compared with the PSG for all sleep-wake stages (Fig. 2a, Table 2). Graphed sleep profiles showed several false-positive and false-negative identifications of REM periods when comparing the behavior-based criteria to PSG (Fig. 2b). However, following ATC treatment of levodopa at 4 mg/kg, hemiparkinsonian rats showed good levels of agreement (CCC <0.8) between the behavior-based criteria compared with PSG for all sleep-wake stages (Fig. 2c, Table 2). Graphed sleep profiles also showed close matches in the sleep-wake stage classification (Fig. 2d).

**Table 1:** Concordance correlation coefficient (CCC) and 95% CI measuring agreement between PSG and behavior-based criteria and between two blinded raters in control rats.

Sleep-wake stage	Comparison	Concordance correlation coefficient (CCC)	95% Confidence interval (CI)
% Wake	PSG vs. Behavior-based criteria	0.984	0.938, 0.996
	Scorer 1 vs. Scorer 2	0.943	0.819, 0.983
% NREM	PSG vs. Behavior-based criteria	0.983	0.934, 0.996
	Scorer 1 vs. Scorer 2	0.922	0.769, 0.975
% REM	PSG vs. Behavior-based criteria	0.971	0.906, 0.991
	Scorer 1 vs. Scorer 2	0.856	0.589, 0.955

In control rats (*n* = 10), there was good agreement between the polysomnography (PSG) vs. the behavior-based criteria and between the two blinded raters (all CCC >0.8). Behavior scores for comparison against PSG were obtained using the average scores of the two raters. These results are demonstrative of the of the face-validity and inter-rater reliability of the criteria

**Table 2:** Concordance correlation coefficient (CCC) and 95% CI measuring agreement in untreated and levodopa-treated hemiparkinsonian between PSG and behavior-based criteria.

Sleep-wake stage	Comparison	Concordance correlation coefficient (CCC)	95% Confidence interval (CI)
Untreated hemiparkinsonian rats			
% Wake	PSG vs. Behavior-based criteria	0.156	−0.285, 0.543
% NREM	PSG vs. Behavior-based criteria	0.294	−0.344, 0.747
% REM	PSG vs. Behavior-based criteria	0.267	−0.263, 0.673
Levodopa-treated hemiparkinsonian rats			
% Wake	PSG vs. Behavior-based criteria	0.967	0.77, 0.996
% REM	PSG vs. Behavior-based criteria	0.961	0.744, 0.995
% REM	PSG vs. Behavior-based criteria	0.997	0.964, 1

In untreated hemiparkinsonian rats (*n* = 5), there was poor agreement between the polysomnography (PSG) vs. the behavior-based criteria for all sleep-wake stages (all CCC <0.8). However, in levodopa-treated hemiparkinsonian rats (*n* = 4), there was good agreement between the PSG vs. the behavior-based criteria for all sleep-wake stages (all CCC >0.8). The behavior-based criteria were implemented by a blinded scorer for each group. These results are demonstrative of the effect of levodopa treatment on improving sleep-wake stage classification

### Video recordings show qualitative improvements in hemiparkinsonian rat sleep-slouch

Evaluation of video recordings of control and hemiparkinsonian rats showed qualitative differences in the *sleep-slouch*, a unique and characteristic behavior that occurs during the onset of REM sleep. In control rats, the sleep-slouch was seen in real time (at normal playback speed) as it occurred quickly and the drop of the animal’s body due to gravity was observed. In contrast, in hemiparkinsonian rats, the sleep-slouches were subtle and cannot be seen in real time as they occur slowly and are therefore less discernible. A closer examination of the video recordings showed that while behaviors characteristic of REM sleep are preserved in hemiparkinsonian rats, the quality of these behaviors became more subtle as reflected in a decreased accuracy in sleep-classification using the behavior-based criteria. However, following levodopa treatment, the sleep-slouch qualitatively improved in hemiparkinsonian rats, occurred over faster timescales, was clear and was comparable to those seen in the control rats (Supplementary Video 1).

### Acute ATC dosing of levodopa improves RSWA in hemiparkinsonian rats

**Fig. 3:**
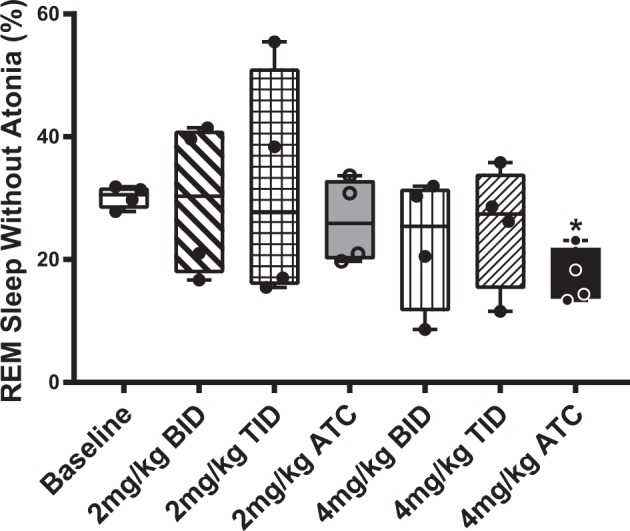
Comparison of mean percentages of tonic REM epochs with positive muscle activity (RSWA). There was a statistically significant decrease in the percentage of tonic REM epochs with positive muscle activity (representative of RSWA) using the 4 mg/kg ATC dosing (*p < 0.05 vs. baseline) in the levodopa-treated hemiparkinsonian rats (*n* = 4). This decrease was not seen with any other dosing regimen (BID—two times per day, TID—three times per day, ATC—around-the-clock every 4 h for 24 h). Box plot shows minimum and maximum ranges and all data points. Error bars indicate ± s.e.m.

ATC dosing of levodopa at 4 mg/kg in hemiparkinsonian rats (17.3 ± 2.2) compared to baseline (30.2 ± 0.9) resulted in a significant decrease in RSWA (*F* (3, 18) = 4.543, *p* = 0.0398) (Fig. 3). In contrast, no statistically significant differences were observed with 2 mg/kg two times per day (BID) (29.6 ± 6.3), 2 mg/kg three times per day (TID) (31.5 ± 9.5), 2 mg/kg ATC (26.3 ± 3.4), 4 mg/kg BID (22.8 ± 5.3), and 4 mg/kg TID (25.5 ± 5.0) dosing regimens compared to baseline. Electrooculograph (EOG) was used to confirm the RSWA observed and used to differentiate the tonic and phasic REM epochs.

### Levodopa dosing regimens did not cause changes in overall sleep-wake states

**Fig. 4:**
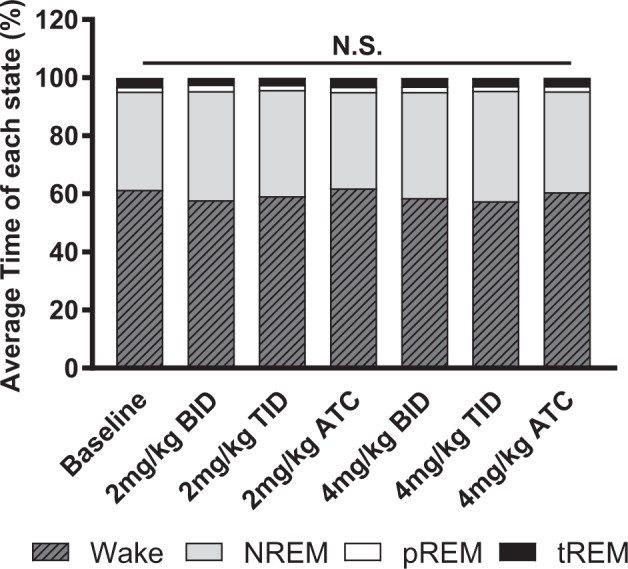
Comparison of average time in Wake, NREM, phasic REM, and tonic REM epochs across differing dosing regimens. There were no statistically significant differences in any sleep-wake stage (Wake, NREM, phasic REM (pREM), and tonic REM (tREM)) irrespective of the dosing regimen used. This suggests that the differential number of arousals caused by various drug regimens did not alter the overall sleep architecture in the treated hemiparkinsonian rats (*n* = 4). (N.S.—nonsignificant, BID—two times per day, TID—three times per day, ATC—around-the-clock every 4 h for 24 h).

Comparison of the mean percentages of all sleep-wake states (Wake, NREM, phasic REM, and tonic REM) between baseline and the six different levodopa dosing regimens showed no statistically significant differences (*F* (6, 18) = 0.6305, *p* = 0.7042). This suggests that the arousals caused by variability in number of injections used to administer levodopa did not cause any significant alterations to the overall sleep architecture in hemiparkinsonian rats (Fig. 4).

### ATC dosing of levodopa improves impaired limb motor functions in hemiparkinsonian rats

**Fig. 5:**
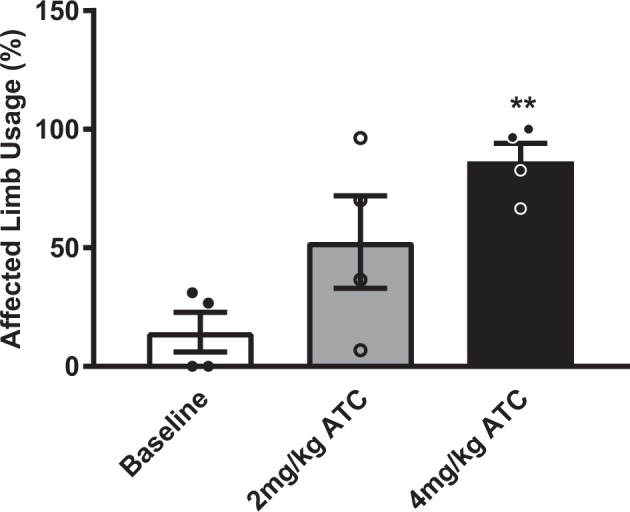
Effects of levodopa treatment on motor symptoms measured by the vibrissae-evoked forelimb placement test. The affected limb usage measured using the vibrissae-evoked forelimb placement test showed a significant alleviation of parkinsonian motor deficits at the 4 mg/kg ATC dosing (**p < 0.01 vs. baseline) compared to post-lesion baseline scores in levodopa-treated hemiparkinsonian rats (*n* = 4). The 2 mg/kg ATC dosing showed a trend toward improvement that did not reach levels of statistical significance. Data are expressed as mean ± s.e.m. (ATC—around-the-clock every 4 h for 24 h).

The vibrissae-evoked forelimb placement following 4 mg/kg ATC dosing (86.4 ± 7.5) compared to the post-lesion baseline (14.4 ± 8.3) showed a significant improvement in affected limb usage (*F* (3, 6) = 2.468, *p* = 0.0075). The 2 mg/kg ATC (52.4 ± 19.5) dosing showed an upward trend but did not reach levels of statistical significance (Fig. 5). The unaffected forelimb usage showed no changes in usage in any rats. The development of drug-induced dyskinesias interfered with the ability to conduct the vibrissae-evoked forelimb placement test in hemiparkinsonian rats during the BID and TID testing drug regimens and were therefore excluded from analysis. Control rats did not show any deficits on the test (data not shown).

### Hemiparkinsonian rats show a profound unilateral loss of nigrostriatal neurons

**Fig. 6:**
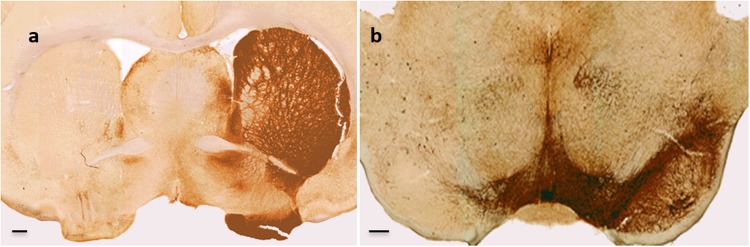
Representative photomicrographs of tyrosine hydroxylase immunohistochemistry from hemiparkinsonian rats. **a** Mid striatal coronal section showing nearly complete dopaminergic denervation of the left striatum and intact nigrostriatal innervation of the right striatum. **b** Section through the substantia nigra demonstrating near complete denervation in the lesioned (left) side and an intact contralateral (right) side. Scale bar: 500 µm.

Histological examination of hemiparkinsonian rats showed greater than 95% depletion of dopaminergic neurons in the substantia nigra on the lesioned side (Fig. 6b) and a dramatic loss of tyrosine hydroxylase positive fibers in the corresponding striatum (Fig. 6a) as expected from this PD model. Unbiased stereological estimations on the right substantia nigra and Abercombie corrected manual counts on the left substantia nigra confirmed these histological observations. All rats used in this experiment met the criterion of 95% depletion of nigral neurons. No evidence of histological lesions in the locus coeruleus, raphe nuclei, sublaterodorsal nucleus, or any areas that have been described previously in models of RBD was found.^27^

## Discussion

To our knowledge, this is the first study of the effect of ATC administration of levodopa on RSWA in the well-studied hemiparkinsonian rat model. Our data provides evidence that RSWA in the hemiparkinsonian rat model of PD is levodopa responsive if dosed ATC and that such dosing does not result in overall sleep-wake architecture changes. Additionally, we describe here a well-characterized behavior-based criteria that helps classify sleep-wake stages in both control and treated hemiparkinsonian rats that exhibit RSWA with accuracy comparable to that of the gold standard of PSG. Further, we observe behavioral changes in hemiparkinsonian rats, during REM sleep which could be potentially combined with PSG to provide a better understanding of RBD-like behavior in PD rodent models. The sleep-slouch posture described here is a significant marker of the onset of REM sleep and could potentially parallel akinesia observed during REM sleep. This posture was less robust and slower in lesioned rats compared to unlesioned control rats reflecting a lack of atonia as a consequence of parkinsonism. ATC levodopa treatment of hemiparkinsonian rats led to the restoration of the sleep-slouch to normal levels suggesting that RWSA is responsive to dopamine replacement therapy in this model of PD.

RBD is one of the most understudied aspects of the sleep-wake alterations in animal models of PD (see review in ref. ^28^). The unilateral 6-OHDA lesioned hemiparkinsonian rat is a well-described preclinical model of PD that has been extensively used for obtaining critical preclinical safety and efficacy data for all PD medications in contemporary use.^29^ This animal model has also led to a better understanding of the pathophysiology of basal ganglia dysfunction in PD and is increasingly recognized as a useful model to study many of the non-motor features of PD including sleep dysfunctions in PD. Other rat models using intracerebroventricular or bilateral striatal injections of 6-OHDA have also shown a dysregulation of circadian mechanisms caused by a disruption of dopamine signaling.^30,31^ However, such models also cause debilitating disabilities, significant morbidity, and variable mortality, which may contribute to the sleep deficits seen.^31,32^ The rotenone and cycad-induced PD rat models mimic sleep disturbances seen in humans prior to the onset of clinical symptoms of PD, but produce exceedingly mild locomotor dysfunction that are both bilateral and symmetric, unlike what is observed in human PD.^33,34^ Similarly, intranasal or direct intranigral administration of lactacystin, a natural proteasome inhibitor fails to produce stable parkinsonism while causing RBD in the rat.^35^ While several studies have shown that inactivation of the glutamatergic neurons of the sublaterodorsal nucleus in rat models results in RSWA,^36–39^ the occurrence of RBD is most often seen in the early stages of PD when there is very little evidence of damage to this region. Moreover, the role of nigrostriatal degeneration, the dominant pathophysiology seen in PD, on RBD associated with PD is not explained by these models. In contrast, the hemiparkinsonian rat model displays this hallmark degeneration of the nigrostriatal dopaminergic pathway and the unilaterality of motor deficits, which can be characterized using well-established tests that have been extensively validated. Since PD always begins with unilateral symptoms (stage I disease), the 6-OHDA lesioned hemiparkinsonian rat simulates this natural disease progression as well. Crucially, we have previously shown that the sleep architecture in this rat model resembles that of PD patients and may thus be useful to elucidate the pathophysiological basis of PD sleep disturbances and be useful to test experimental therapies for RBD in early PD.^22^

While some rudimentary species-specific sleep postures and behaviors have been reported previously, most pre-clinical models of PD lack a codified rodent equivalent of human behaviors that are commonly associated with RBD.^40–44^ This issue is exacerbated by the lack of standard PSG recording procedures and sleep stage classification in stark contrast to the well-accepted methods commonly applied to human sleep studies.^45^ Our paper provides such a well-defined behavior-based criterion for sleep-stage classification in rodents with time-locked PSG recordings that has been validated and tested with experimental therapeutics. A review of sleep staging systems in rats^21^ indicate that with few exceptions^46,47^ most sleep studies classify REM sleep as a single stage which could lead to an inaccurate classification of RSWA. In contrast, the use of EOG recordings and a stringent two standard deviation definition for positive muscle activity in the present study allows for the bifurcation of REM sleep into its tonic and phasic components and consequently accurately identify RSWA. Further, the use of a behavior-based criteria in conjunction with polysomnography recordings is also in line with newer scales used to detect and quantify RBD in humans such as the REM Sleep Behavior Disorder Severity Scale.^48^

There is a plethora of evidence that shown that the dopaminergic neurons present in the substantia nigra pars compacta (SNpc) play an important role in the regulation of REM sleep in rats.^49–51^ These neurons receive inputs from the locus coeruleus (REM-OFF) and laterodorsal/pedunculopontinetegmentum (REM-ON), neurons which could modulate the presence of RBD.^52^ Hemiparkinsonian rats also show reduced ratios of distance and transitions between the resting vs. the activity phase, which could potentially parallel similar abnormalities seen in RBD associated with PD.^36^ A variety of underlying mechanisms including dopamine release from the unlesioned hemisphere, disruptions in the mesopontine dopaminergic neurons, and excessive nigral GABAergic inhibition via the pedunculopontine nucleus (PPN) have been proposed for these REM sleep disturbances seen in hemiparkinsonian rats.^49,53–56^ Number of studies also suggest that divergent midbrain dopaminergic populations have different effects on sleep-wake regulation and RSWA. Dopaminergic cells that originate in the ventral tegmental area and the dorsal raphe have been implicated as key arousal promoting systems, which are counterbalanced in health by SNpc dopaminergic neurons that promote sleep.^57^ The lesioning of SNpc dopaminergic neurons may therefore create an imbalance that contributes to RSWA and the RBD-like features in the hemiparkinsonian rat model. In spite of several lines of evidence indicating a dopaminergic basis for the development of RBD in PD, few studies have analyzed the effects of dopamine replacement therapy on this sleep dysfunction in the context of a preclinical model of PD.

Studies have suggested that the presence of RBD in PD may put patients at higher risk for levodopa-induced motor fluctuations in wakefulness.^58–61^ This finding may indicate that PD patients with concomitant RBD have a more severe form of neurodegeneration or that RBD is an at-risk biomarker for motor fluctuations in PD. Recent studies have proposed that many of the deleterious effects of levodopa treatment can be minimized by modifying dosing regimens to provide continuous dopaminergic stimulation.^62–64^ The varying regimens in the current study were formulated based on pharmacodynamics of levodopa to test the effects of intermittent oscillating versus continuous non-oscillating dosing schedules. Fluctuations in levodopa concentrations caused by conventional BID and TID dosing regimens have been shown to result in wider oscillations in striatal dopamine availability causing profound pathophysiological alterations in the basal ganglia which in turn cause motor complications and RBD.^65–67^ We show here that ATC dosing regimen at 4-h intervals potentially overcomes the issue of oscillating levels of striatal dopamine and thereby mitigates daytime motor deficits and night time RSWA. The relationship between these two beneficial effects of ATC levodopa dosing to mitigate PD-related phenomenology requires further study.

Our observations are further strengthened by recent clinical studies where levodopa/carbidopa intestinal gel was administered via intrajejunal pump continually for 24 h.^25,68,69^ In these patients, there was a significant overall improvement in their sleep quality as measured by the Parkinson’s Disease Sleep Scale and Non-Motor Symptoms Scale consistent with improvement of nocturnal akinesia.^69^ Studies also show that sleep abnormalities are not adequately addressed with optimized daytime dosing of medication and that untreated sleep disturbances have alarming consequences for daytime function in PD patients. New treatment approaches in treating PD patients therefore, recommend the utilization of 24-h continuous treatment strategies used here to sufficiently manage and control both daytime and nighttime symptoms.^70–72^ Advances in formulations and routes of administration have allowed for such treatments in PD patients and suggest that ATC dopamine agonist therapies may provide benefits to subjective measures of sleep.^73^ Recent studies of rotigotine, a highly lipophilic dopamine-receptor agonist delivered via transdermal patches has been shown to improve subjective sleep quality in PD patients with RBD^74^ and was found to be effective in both early^75^ and advanced stage PD patients.^76^ The dopamine agonist, ropinirole in its 24-h prolonged release formulation has been found to efficacious in improving nocturnal symptoms of PD in double-blind, placebo-controlled trials.^77,78^ Similarly, intrajejunal infusions of levodopa–carbidopa intestinal gel (Duodopa) has also been shown to improve sleep quality in PD patients.^69,79^ These new advances in routes of administration lead to 24-h, continuous, nonfluctuating drug levels and may therefore help ameliorate RBD in PD.

There are some drawbacks to our study that need to be addressed. Only a low-to-medium range of levodopa doses were tested in the current study. This is in keeping with the dose of levodopa that is typically employed in early PD patients. Testing higher dosages of levodopa may be useful to further clarify its effects on RBD in future studies. Though animals had well-established baseline recordings and a washout period between dosages of LD our study lacks a cohort of vehicle injected animals. However, our before and after study design does provide the proof of principle to undertake additional longer studies that include separate control groups. Studies that include longer segments of PSG and video recordings with multiple camera angles may have additional benefits and help further elucidate the behavior-based criteria described here. Future studies that utilize osmotic minipumps or other methods for continuous levodopa delivery as shown in a recent study^80^ may be utilized to further test the feasibility of our ATC levodopa therapeutic approach. The behavior-based criteria reported here requires well-trained and experienced raters and is labor intensive. Many hours of training were needed for the raters to learn the behavior-based criteria. Future studies could also try to implement the use of computational software to allow for automatic classification that will permit ease of use. Finally, the use of telemetric implantable devices that permit the use of cardiac, respiratory, and jaw muscle group monitoring were not available for polysomnographic classification in the current study and may have added advantages in future studies.

In conclusion, our findings support the dopaminergic mechanisms for RBD, an important non-motor aspect of PD. The characterization of RBD as a non-motor aspect of PD does represent a paradox, despite its continued characterization as such in most PD literature. This is because, movement and tone abnormalities are the key aspects of RBD, and they are indeed motor phenomena albeit occurring during sleep. Our study in the hemiparkinsonian rat (which has exclusive lesioning of the nigrostriatal pathway with preservation of other relevant monoamine systems due to the use of desipramine during lesioning) suggests that the nigrostriatal dopaminergic pathway may play a seminal role in RBD and sleep disturbances seen in this PD model as reported elsewhere.^51,81,82^ Our findings suggest that dopamine dysregulation contributes to RSWA and that normalizing this with ATC dopaminergic replenishment mitigates this sleep abnormality. Our results show that ATC levodopa dosing modulates REM sleep disturbances much better than intermittent BID or TID dosing. The use of ATC dopamine administration and its ability to provide non-fluctuating levels of dopamine replenishment could help cover both daytime and nighttime symptoms of PD. The advent of new drug-delivery systems such as intra-jejunal levodopa gel infusion therapy and transdermal dopaminergic patches could translate these findings to benefit PD patients. We also propose behavior-based criteria that resembles behavior rating scales for RBD in humans and could help facilitate studies into the mechanisms underlying RBD in PD using this animal model. Since RBD could potentially be a harbinger of PD, this animal model-based criteria could have broad applicability for the development of effective neuroprotective therapies.

## Methods

Twenty-two female Sprague–Dawley rats weighing 220–250 g at the time of acquisition and housed on a 12-h light:dark cycle (8 am–8 pm) with ad libitum access to food and water were used in the experiment. All procedures were carried out in accordance with the guidelines in the NIH Guide for the Care and Use of Laboratory Animals and were approved by the Pennsylvania State University Institutional Animal Care and Use Committee. A set of normal rats were used to create the behavior-based criteria (*n* = 3) and the remaining rats were randomly assigned to either the hemiparkinsonian (*n* = 9) or unlesioned control (*n* = 10) groups. Rats in the hemiparkinsonian group were further randomly subdivided into an untreated (*n* = 5) and levodopa-treated (*n* = 4) groups.

### Lesioning surgery

Hemiparkinsonism was induced in nine rats by lesioning the left nigrostriatal pathway using a modified version of the Ungerstedt model.^83^ Briefly, 6-hydroxydopamine-hydrogen bromide (12 μg in 4 μl) was stereotactically injected into the medial forebrain bundle (antero-posterior (AP): −1.5 mm, medio-lateral (ML): +1.8 mm, dorso-ventral (DV): −7.5 mm from bregma, the midline suture and the skull surface, respectively) at a rate of 0.67 μl/min using a gastight Hamilton syringe, following which the needle was left in place for 5 min. Prior to the surgery, rats received an injection of the noradrenergic uptake blocker desipramine (15 mg/kg i.p.) to avoid damage to norepinephrine (NE) pathways and to limit the lesion effect to the SNpc in one hemisphere. After 3 weeks of recovery, the lesioned rats were challenged twice, 2 weeks apart with apomorphine hydrochloride (0.2 mg/kg, s.c.). The apomorphine-induced rotations were counted in an automated ‘rotometer’ (San Diego Instruments). Rats with more than 245 rotations over 35 min on two separate sessions were classified as hemiparkinsonian rats and used in the study.^84^ Ten rats did not receive the lesioning surgery and were used as controls.

### Electrode implantation surgery

Subsequently, all rats were implanted with stainless-steel skull screws over the frontal and parietal cortices for electroencephalograph (EEG) recordings, a pair of wire electrodes in the nuchal muscles for electromyograph (EMG) recordings and two screws in the supraorbital ridge for electrooculograph (EOG) recordings under general anesthesia as described previously.^85,86^

### Polysomnography recordings

After recovery from surgeries, polysomnography (PSG) comprising of EEG, EMG, and EOG recordings was obtained, analyzed, and averaged to obtain equal “lights-on” and “lights-off” recording times for all rats. Signals were amplified and filtered with a Grass Model 12 Neurodata amplifier system (Grass Instrument Division of Astro-Med, Inc., West Warwick, RI), and digitized at 128 Hz with a USB-2533 16-bit analog to digital converter (Measurement Computing Corporation, Norton, MA). The EEG, EMG, and EOG signals were filtered offline in Matlab (Mathworks), segmented into 10-s epochs, and manually rated using the commercial SleepWave program (Biosoft Studio, Hershey, PA). The behavior criteria development relied on EEG and EMG recordings alone to classify each 10-s epoch as demonstrative of wakefulness, NREM sleep, and REM sleep.^22^ Subsequently, to study RSWA, the REM sleep component was further divided into tonic REM sleep and phasic REM sleep using EOG recordings (described below).

### REM sleep classification

**Fig. 7:**
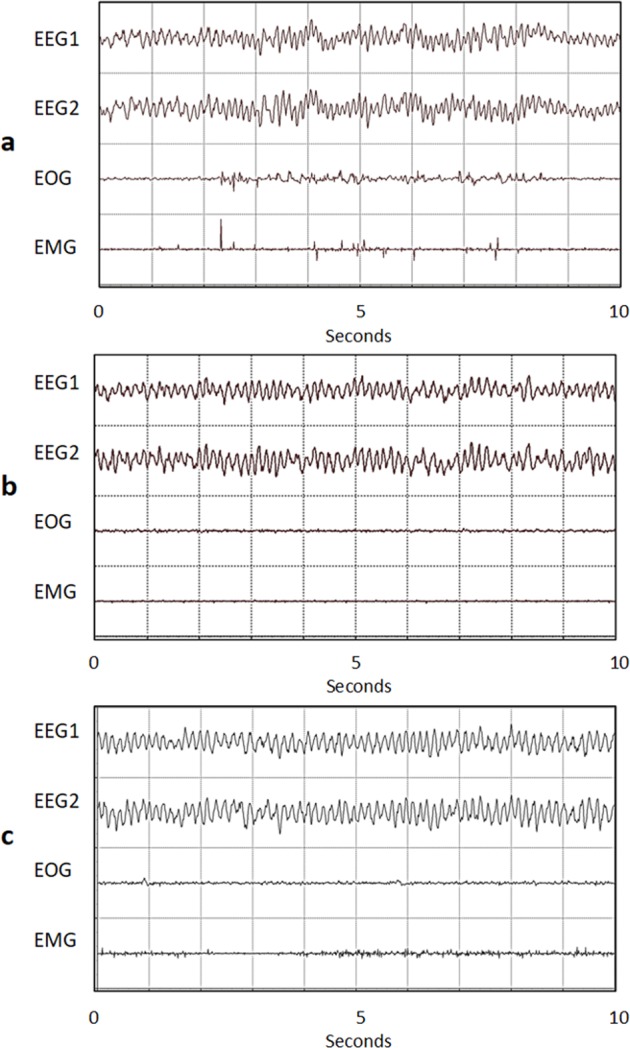
Polysomnography patterns of phasic and tonic REM of an hemiparkinsonian animal, including 2 EEG, 1 EOG, and 1 EMG channels. **a** Muscle activity during phasic REM, characterized by presence of EOG and EMG activity (normal); **b** Atonia during tonic REM, characterized by lack of EOG and EMG activity (normal); **c** Muscle activity during tonic REM, or REM sleep without atonia (RSWA), characterized by the lack of EOG activity and presence of EMG activity, which is representative of RBD in rats.

Baseline PSG recordings (24 h each) were obtained from all rats and were aligned to the 12-h light/dark cycles after which the rats received levodopa (described below). The rats were then recorded to obtain post levodopa PSG. Sleep-wake classification was carried out using only EEG and EOG recordings. EOG recordings were further used to differentiate REM sleep into its phasic (characterized by bursts of EOG spikes) and tonic components (characterized by periods of little or no EOG activity components) (Fig. 7). EMG was used to evaluate for positive muscle activity, defined as muscle activity during tonic REM sleep that was two standard deviations above the baseline atonia. The percentage of tonic REM with positive muscle activity was determined and compared between baseline and each levodopa dosing regimen.

### Levodopa dosing regimens

All levodopa-treated hemiparkinsonian rats (*n* = 4) received injections of levodopa and benserazide, on separate occasions at two different doses, 2 mg/kg or 4 mg/kg, of levodopa and 15 mg/kg benserazide (i.p.). Benserazide has been shown to inhibit peripheral aromatic l-amino acid decarboxylase (AADC) activity, enhance brain bioavailability of levodopa, and increase extracellular striatal dopamine levels in hemiparkinsonian rats.^87^ Microdialysis studies in unlesioned rats^88–91^ and hemiparkinsonian rats^87,92^ show that levodopa plus benserazide significantly increases levels of extracellular dopamine in the striatum for up to 4 h post injection compared to baseline levels. Three different dosing regimens were utilized: BID, TID, and ATC every 4 h for 24 h (Supplementary Fig. 1). The first injection of each dosing regimen was aligned with the lights-on time of 8 am. The BID levodopa injections were administered at 8 am and 4 pm. The TID levodopa injections were administered at 8 am, 12 pm, and 4 pm. Finally, the ATC levodopa injections were administered at 8 am, 12 pm, 4 pm, 8 pm, 12 am, and 4 am. The overall daily dosage of levodopa that each animal received was constant and was sub-divided into parts depending on the dosing regimen being used. The BID and TID doses were given only during the 2-h lights-on phases as rats were more likely to be in REM sleep. All rats in the levodopa treatment group received 2 days of injections of all dosing regimens, which were separated by washout periods of 24 h to analyze the effects of each one independently. PSG recordings were obtained for all sessions spanning about 48 h of recordings from each rat for each dosing regimen. All rats were acclimatized to injections prior to the start of any experimentation and after were continually observed after each injection until they resumed their previous behavioral state.

### Behavior-based criteria development

To create a set of behavior-based criteria, video recordings were time-locked with PSG recordings of a cohort of normal rats (*n* = 3). Video recordings were then reviewed and the behaviors of the animals in each stage were recharacterized by a blinded rater. Video observations and complementary PSG recordings of rats showed six distinct behaviors characterizing the three sleep-wake stages (Table 3). *Wakefulness* was characterized by behaviors including arousal posture (bipedal activity, eating, posture of the extended tail, whisking, and grooming); *NREM* was characterized by closed eyes, stationary position, tail not extended, and chin resting on tail or ground; and *REM* was characterized by the *sleep-slouch*. The *sleep-slouch* was a movement of the rat’s body, head, or both to one side of the body caused by gravity rather than active muscle contraction (Supplementary Video 1). This phenomenon served as a behavioral correlate of REM sleep and was followed by RSWA in hemiparkinsonian rats. A score (Table 3) was assigned to each behavior in correlation with PSG that resulted in a total score at any time point denoting the current sleep-wake stage of the rats.

**Table 3:** Six signs comprising the behavior-based criteria for sleep-wake stage classification.

Behavioral-based criteria: Signs	Score
Arousal posture: Moment of temporary wakefulness characterized by quick jerky motions indicative of muscle contractions, extension of a limb, rising of the head, and elevation of the back	−2
Eyes closed: The black of the rat’s pupil is completely covered by the eyelids such that the top and bottom lids are touching	1
No movement for a period of ≥5 s: The entire body remains still for a period of ≥5 s	1
Tail not extended: The tail is coiled around the body such that the tip of the tail is anterior to where the tail exits the body. The angle at which the tail leaves the body is <90°. The tail must be touching the body at a point anterior to the root of the tail	1
Chin resting on tail or ground: The chin is reclining on the ground, tail, or on another structure so as to not be elevated	1
Sleep-slouch posture: Movement of a rat’s body, head, or both that occurs during sleep, caused only by gravity acting on a part of the body, not by contraction of muscle	3

Scoring for the behavior-based video criteria used the following rubric: ≤1: indicates wakefulness, 2–4: indicates non-REM (NREM) sleep, and ≥5: indicates REM sleep

### Behavior-based criteria vs. PSG recordings and inter-rater agreement

To test the behavior-based criteria against the gold standard of PSG, hour-long video recordings from the lights-on periods of control rats were scored by a blinded rater (Scorer 1) using the behavior-based criteria. A second rater (Scorer 2) was then used to test the inter-rater reliability of the behavior-based criteria. The percentage of time spent in each sleep-wake stage was calculated and averaged between the Scorer 1 and Scorer 2, then compared to that obtained from EEG/EMG alone without the use of EOG recordings. To determine whether any agreement is reflective of the actual sleep-wake patterns, the sleep profiles were graphed and compared to those obtained from PSG. In all graphing of the stages of wakefulness, the stages were split into 10-s epochs with REM receiving the highest priority in the instance of an epoch containing more than one sleep-wake stage.

This comparison with PSG was again performed on the hemiparkinsonian and the levodopa-treated hemiparkinsonian rats using a blinded rater. Given the unreliability of EMG in the presence of RSWA-like behaviors in hemiparkinsonian rats (movement artifacts), this comparison was performed using the EEG and EOG recordings against the rater for the untreated hemiparkinsonian group and the levodopa treated hemiparkinsonian group. CCC with 95% CI was calculated to assess agreement i.e.reliability for all comparisons. The level of probability for good agreement was set at a CCC >0.8.

### Vibrissae-evoked forelimb placement test

To evaluate the efficacy of levodopa treatment on forelimb motor deficits, rats underwent the vibrissae-evoked forelimb placement test^93^ at the end of each dosing regimen. Previously, we have shown that hemiparkinsonian rats show profound deficits on this test after lesioning and that these deficits were ameliorated by levodopa/benserazide treatment.^94^ Since testing in the current study was performed during the lights-on period when the animals are less active, we chose this evoked test as a measure of forelimb function. The rats were tested immediately following one of the last injections to test the drug at maximum efficacy and separated from any polysomnographic recordings that were utilized for analysis from these animals. Briefly, animals were held at the torso so that the hind limbs and the forelimb being tested could hang freely while the forelimb not being tested was carefully restrained. The unrestrained forelimb was evaluated by bringing the rat toward the edge of the tabletop to elicit an evoked reaching behavior toward the surface due to stimulation of the ipsilateral vibrissae contact with the table surface. This response was tested for each forelimb independently for ten trials and repeated three times. The number of successful placements for each forelimb onto the tabletop was scored to determine the percentage of affected forelimb usage.

### Histology

At the end of the study, all rats were euthanized via transcardiac perfusion with cold heparinized saline and 4% paraformaldehyde fixative. Brains were removed, cryoprotected, and sectioned coronally at 60 µm. Sections were processed for cresyl violet (Nissl stain) and tyrosine hydroxylase immunohistochemistry to verify unilateral depletion of the nigrostriatal pathway as described previously.^94^

### Stereology

Estimates of tyrosine hydroxylase positive neurons in the substantia nigra was performed using a design-based optical fractionator method (Stereo Investigator, MBF Bioscience) on the unlesioned right substantia nigra, using systematic random sampling and unbiased stereological estimation. The Abercrombie correction to manual physical counts was applied when counting procedures did not meet the criteria for the optical fractionator as was the case with the lesioned left substantia nigra.^95^

### Statistical analysis

All statistical analysis was performed by an investigator who was blinded to all experiments on animals, polysomnographic and behavioral raters to avoid any bias (A.K.). For additional rigor, histological analysis and stereology was performed by investigator blinded (K.V.) to all other experimental assessments. CCC with 95% CI was calculated using R software, version 3.5.1 (The R Foundation for Statistical Computing, Vienna, Austria) to assess inter-rater reliability between raters and to assess face validity between polysomnography and the criteria. The level of good agreement was set at a CCC >0.8.

One-way ANOVA followed by Dunnett’s multiple comparisons test was calculated using GraphPad Prism version 7.05 for Windows, GraphPad Software, La Jolla, California, USA, www.graphpad.com, that accounts for the within-animal correlation of multiple levodopa doses per rat, was used for analysis of the behavioral assessment, the percentage of tonic REM epoch with muscle activity between baseline and each levodopa dose and the total REM epoch. Data are expressed as mean ± SEM and the level of probability for statistical significance was set at 0.05.

### Reporting Summary

Further information on research design is available in the Nature Research Reporting Summary linked to this article.

## Supplementary information

Supplementary Information File

Supplementary Video 1

Reporting Summary

## Data Availability

The data used to support the findings of this study are available from the corresponding author upon request.
