# Supplementary material for: Acute levodopa dosing around-the-clock ameliorates REM sleep without atonia in hemiparkinsonian rats
Source: NPJ Parkinsons Dis. 2019 Nov 29;5:27. doi: 10.1038/s41531-019-0096-2 (PMC6884572; doi:10.1038/s41531-019-0096-2)
Supplement: Supplementary file 1 — Supplementary Information File [file 41531_2019_96_MOESM1_ESM.pdf]

Supplementary Material

Supplementary Figure 1: Study Timeline and Levodopa dosing regimens.

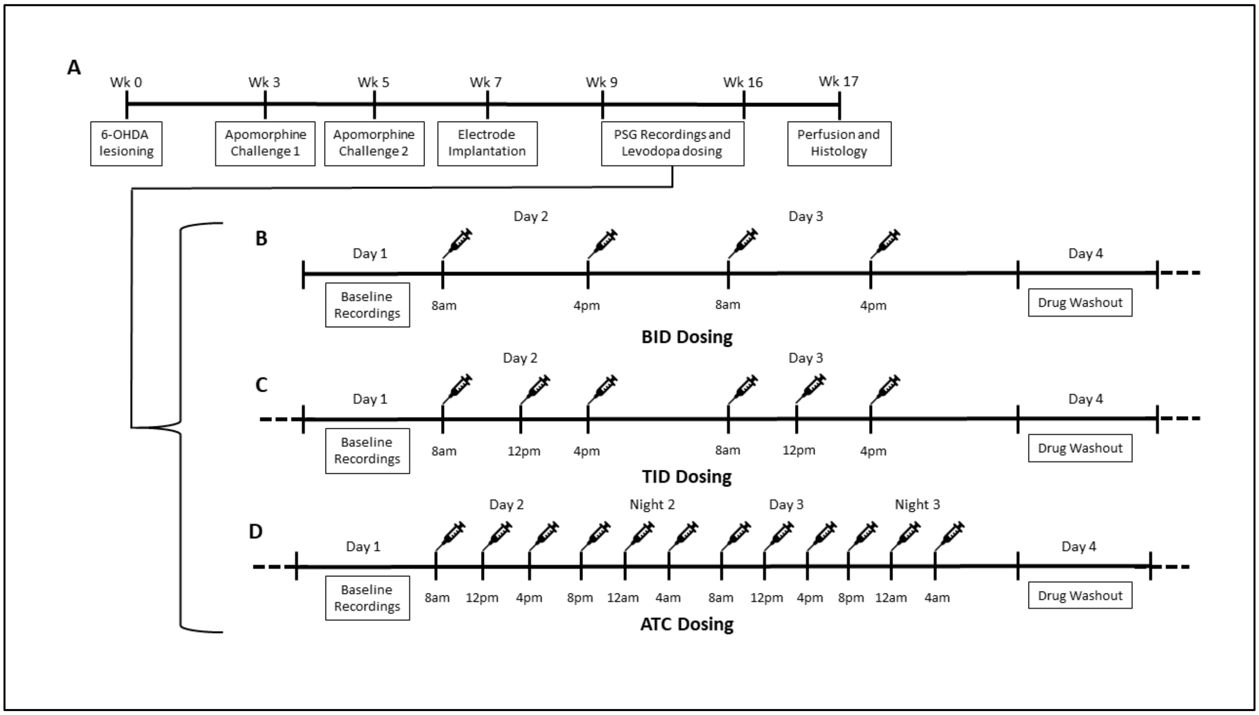

(A) Overall study timeline. Timing of (B) two times per day (BID), (C) three times per day (TID) and (D) ATC every 4 h for 24 h levodopa dosing injections. Each dosing regimen was preceded by 24 h of baseline recordings and followed by 24 h of washout to avoid cumulative dosing effects. All dosing regimens were carried out for both 2mg/kg and 4 mg/kg levodopa dosages.

Supplementary Video 1: Sleep-slouch.

A and B: In control rats, the pronounced drop of the body to one side is observable in real time; C: In hemiparkinsonian rats, the sleep-slouch is not observable in real time; D: In hemiparkinsonian rats, subtle head and body shifts are noted but entire sleep-slouch (not shown) lasted for more than 45-s; E and F: In hemiparkinsonian rats, head and body movements are observable at 16x normal speed; G and H: In levodopa treated hemiparkinsonian rats, both head and body movements are observed in real time though the drop in G is more subtle. Arrows also denote the length of the sleep-slouch.
